# Pesticide and pharmaceutical pollution in South Africa: a review of sources, impacts, and policy gaps

**DOI:** 10.1007/s10661-026-15137-z

**Published:** 2026-03-18

**Authors:** Jessie Mzati Amaechi, Reynold Chow, Leslie Petrik, Nebojsa Jovanovic

**Affiliations:** 1 Department of Earth Science, University of the Western Cape, Bellville, 7535 South Africa; 2 Soil Physics and Land Management Group, Wageningen University & Research, P.O. Box 47, Wageningen, 6700 AA Netherlands; 3 Department of Chemistry, University of the Western Cape, Bellville, 7535 South Africa

**Keywords:** Micropollutants, Regulatory frameworks, Pharmaceutical, Pesticide, South Africa

## Abstract

South Africa faces environmental and public health risks due to pollution of various environmental systems by pesticide and pharmaceutical residues resulting from anthropogenic activities. However, regulatory and monitoring mechanisms remain inadequate. This review aimed to assess occurrences, sources, regulatory frameworks, and policy responses related to pesticide and pharmaceutical pollution in South Africa. The review of published (peer-reviewed) articles and government and policy documents found that pharmaceuticals such as acetaminophen and diclofenac, and pesticides such as atrazine, endosulfan, and chlorpyrifos, are commonly reported micropollutants, even though they are banned. The spatial distribution of the reviews shows that Western Cape, Gauteng, and KwaZulu-Natal appear to have more research conducted on these pollutants. Related laws and policies managed by the Department of Agriculture, Land Reform and Rural Development (DALRRD) and the South African Health Products Regulatory Authority (SAHPRA) are insufficient and lack thorough environmental risk assessments, regular monitoring, and strict enforcement. Comparison with the EU, USA, Switzerland, Australia, Japan, and South Korea reveals that these countries have stronger regulatory systems, including obligatory risk assessments, national take-back schemes, and integrated monitoring, which are mostly absent in South Africa. The informal sale of pesticides, misuse, improper disposal of pharmaceutical waste, and the slow implementation of the Integrated Pest Management (IPM) approach further exacerbate the problem. To prevent future risks to ecosystems and public health, the review recommends regulatory adjustments, improved interagency coordination, and enhanced environmental monitoring systems to align South Africa’s regulatory framework with world best practices.

## Introduction

The use of pharmaceuticals and pesticides has made them common in various environmental media like water (Mugudamani et al., 2023; Oke et al., 2024; London & Rother, 2000; Ndungu et al., 2016), air (Mugudamani et al., 2022), sediments (Buah-Kwofie et al., 2018a; Degrendele et al., 2022a; Mehlhorn et al., 2023), and marine life (Ojemaye & Petrik, 2022; Wolmarans et al., 2021). Studies have also confirmed that micropollutants like pesticides (Gakuba et al., 2019; Horak et al., 2021; Kanan et al., 2022; Navarro et al., 2024) and prescription drugs (Kanan et al., 2022; Kayode-Afolayan et al., 2022; Okoye et al., 2022) cause harm to the ecosystem and danger to human life. In South Africa, the increasing use of pesticides in agriculture, as well as the inappropriate prescribing of prescription drugs, raises concerns about their fate and their impact on the entire ecosystem (Ademoyegun et al., 2020; Degrendele et al., 2022b; Horak et al., 2021). Although pesticides and prescription drugs are important for food security and public health, it is essential to understand their mechanisms, effects, and environmental impacts to develop appropriate laws and policies to ensure ecological safety and sustainability.

South Africa is significantly prone to pollutants due to its large-scale and high-performance agriculture (Dabrowski, 2015; Dabrowski et al., 2014) and the high market demand for prescription drugs (Hodes, 2019; Moodley et al., 2021). Organochlorine and pyrethroid pesticides and prescription drugs, such as antibiotics, are frequently identified in water, sediment, and fish (Fuhrimann et al., 2022; Gakuba et al., 2019; Hatting et al., 2019; Horak et al., 2021; Ojemaye & Petrik, 2022). For example, numerous studies identified the presence of micropollutants, including antibiotics, antiretrovirals, and inflammatory drugs, in some of the rivers of South Africa, such as Umgeni, Msunduzi, and Eerste (Agunbiade & Moodley, 2016). The frequent occurrence and widespread usage of these compounds imply potential ecological harm. The occurrence of these compounds in water bodies may result in water pollution, endocrine disturbances in aquatic organisms, and the development and dissemination of drug resistance (Kayode-Afolayan et al., 2022; Madikizela et al., 2020; Ojemaye & Petrik, 2019, 2022). Numerous studies have indicated that toxic pesticides are present in the ecosystem, which initiates adverse effects on non-target aquatic animals and contributes to the global pesticide resistance threat (Dabrowski et al., 2014; Rauch et al., 2018).

Findings from environmental monitoring indicate that South Africa’s water bodies are exposed to a mix of pollutants, posing complex challenges for risk assessment and management (Chow et al., 2023; Madikizela et al., 2020; Ojemaye & Petrik, 2019). Pharmaceutical pollutants have been shown to exert multiple adverse effects on aquatic life, ranging from biochemical alterations to bioaccumulation, bioconcentration, and biomagnification through the food chain, which may disturb the aquatic ecosystem’s equilibrium. The combined exposure to multiple pesticides also raises concern about how they may affect the system. The impact can affect water quality and the living components of the ecosystem (Chow et al., 2023; Curchod et al., 2020; Mathapelo et al., 2021).

The regulatory environment in South Africa is evolving to address the challenge posed by anthropogenic pollutants, but there are still gaps. Existing regulations usually focus on pesticides, while pollution from pharmaceuticals has largely escaped regulation. This establishes the need for integrated policies covering the entire lifetime of artificial chemicals, from manufacturing and application up to waste disposal and environmental monitoring (Mamera, 2018; Michael et al., 2019). There also remains an additional need for specialised analysis methodologies and monitoring protocols capable of yielding reliable data on contaminants’ presence and behaviour in various environmental matrices (Mamera, 2018; Materon et al., 2020).

In this paper, attempts will be made to consolidate current knowledge of the environmental presence, concentrations, and effects of pesticides and pharmaceuticals on the South African environment. By doing this, gaps in existing knowledge will be identified, and the effectiveness of the regulatory policy needed to restrict the hazards posed by pollutants will be explored. The review is organised under the following headings: (1) Sources of pesticide and pharmaceutical pollution in South Africa; (2) Review of field sampling and laboratory analysis methods for pesticides and pharmaceuticals in South Africa; (3) Review of detected pesticides and pharmaceuticals in South Africa; and (4) Regulations on pesticide and pharmaceutical pollution in South Africa.

## Methodology

### Literature search strategy

This narrative review was carried out using academic databases, including Scopus, Web of Science, Google Scholar, and ScienceDirect. The review focused on peer-reviewed literature published between 2010 and 2025. The longer timeline was chosen to accommodate significant research developments and policy changes in the field. These changes include higher research output in the subject area, driven by increased attention and monitoring. Increased urbanisation, agricultural activities, and pharmaceutical consumption in South Africa have necessitated this attention. For the literature search, keywords and Boolean operators like (“pesticide” OR “pharmaceutical” OR “veterinary drug” OR “antibiotic” OR “hormone” OR “endocrine disruptor”) AND (“pollution” OR “contamination” OR “residue” OR “environmental fate”) AND (“South Africa”) were used.

Relevant policy and regulatory documents were also reviewed. South African national gazettes, legislative frameworks, and key documents from the Department of Water and Sanitation (DWS), Department of Forestry, Fisheries and the Environment (DFFE), Department of Agriculture, Land Reform and Rural Development (DALRRD), and South African Health Products Regulatory Authority (SAHPRA) were also reviewed. For policy comparisons, documents from the EU, USA, Switzerland, Australia, and selected Asian countries were used. For the EU, the consulted documents include Regulation (EC) No. 1107/2009, the Water Framework Directive (2000/60/EC), and Directive 2001/83/EC on medicinal products. For the USA, documents consulted include the Federal Insecticide, Fungicide, and Rodenticide Act (FIFRA), the Food Quality Protection Act (FQPA), and 21 CFR Part 25. For Australia, the Australian Agricultural and Veterinary Chemicals Code Act and guidance from the Australian Pesticides and Veterinary Medicines Authority (APVMA) were examined. Asian documents consulted include regulatory frameworks from Japan’s Ministry of Agriculture, Forestry, and Fisheries and South Korea’s Ministry of Environment.

### Selection and analysis of literature

The search yielded approximately 600 records across all the databases. Duplicates were removed, and further filtering was done based on the relevance of studies to South Africa. We removed or excluded studies that were unrelated to South Africa or fell outside the topic focus, such as those on heavy metals, per- and polyfluoroalkyl substances (PFAS), or mining-related topics. Studies conducted in South Africa that focused on pesticide and pharmaceutical contamination and environmental occurrences in water, sediments, air, and marine biota were included. Also included are studies on regulatory and policy responses to environmental pollutants. Government reports, policy documents, and legislation related to monitoring systems, pollution control, or chemical safety regulation were included. After inclusion and exclusion, the records were reduced to approximately 300, of which 116 were directly reviewed and cited in this paper.

The included literature was then reviewed to extract information, which is organised into sections in this review paper: sources of pesticide and pharmaceutical pollution in South Africa, review of field sampling and laboratory analysis methods for pesticides and pharmaceuticals in South Africa, review of detected pesticides and pharmaceuticals in South Africa, and regulations on pesticide and pharmaceutical pollution in South Africa.

## Sources of pesticide and pharmaceutical pollution in South Africa

**Fig. 1:**
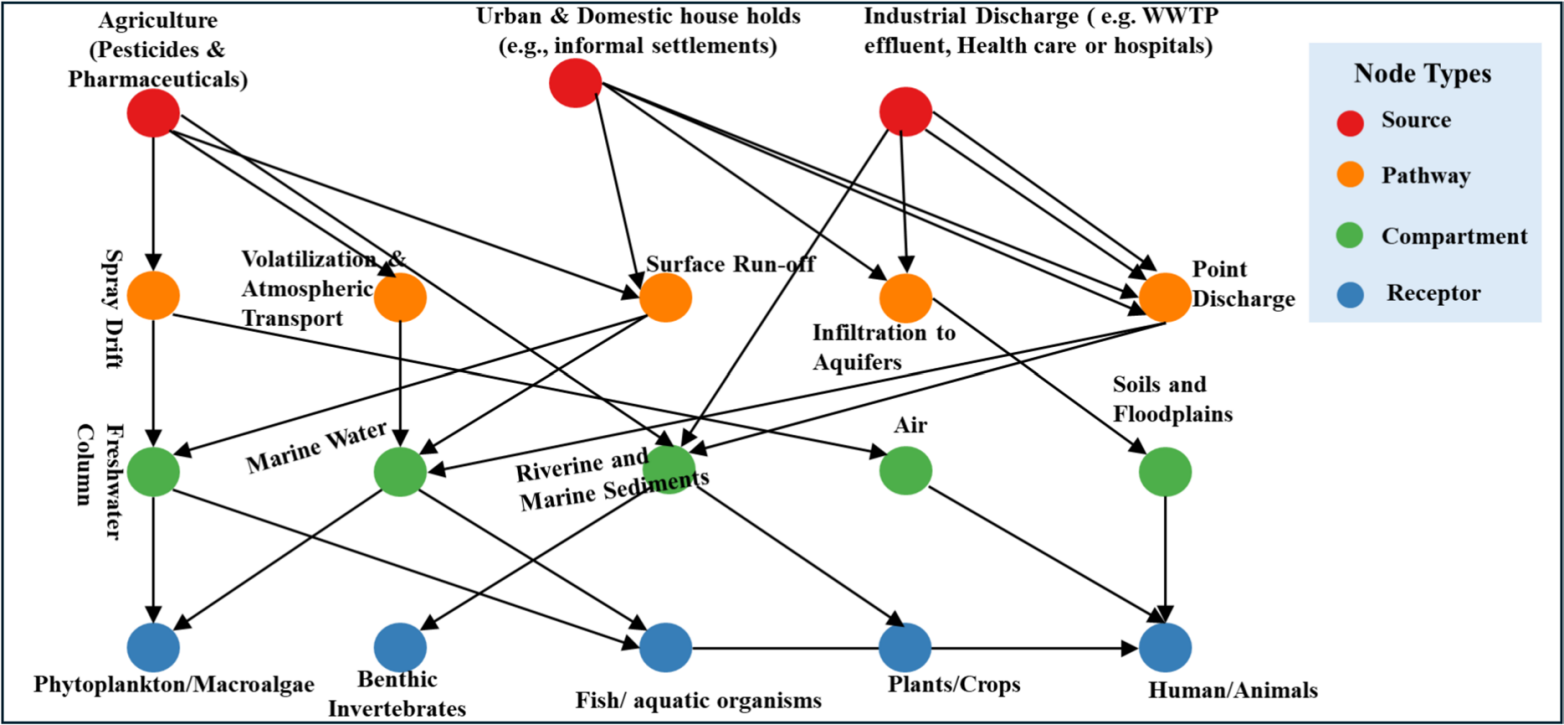
Idealised visual model illustrating the relationship between nodes depicting sources, pathways, compartments, and receptors of pesticide and pharmaceutical pollution

Pesticide and pharmaceutical pollution in South Africa originates from several interlinked anthropogenic activities. These include agricultural production, domestic usage and disposal, industrial manufacturing, healthcare operations, and failure in wastewater and sanitation systems. Socio-economic patterns, land-use practices, regulatory gaps, and infrastructure limitations shape the entry of these substances into the environment. Figure 1 summarises sources, pathways, compartments, and receptors for anthropogenic pollutants in the environment. Research shows that farming and wastewater treatment systems are the most significant sources of micropollutants in the environment. Some of these micropollutants, such as antibiotics, can come from both urban and agricultural use, with additional contributions from homes, hospitals, and informal areas without sewage systems (Chow et al., 2023; Davies et al., 2024; Fuhrimann et al., 2021; Horn et al., 2022a; Rakonjac et al., 2023).

### Agricultural practices

Agricultural activities are thought to be the primary source of pesticide contamination in the South African environment, given that it is one of the highest users of pesticides in sub-Saharan Africa (Dabrowski et al., 2014). Pesticides such as chlorpyrifos (organophosphate), cypermethrin (pyrethroid), imidacloprid (neonicotinoid), and atrazine (triazine) are widely applied to manage crop pests and increase crop yields. These compounds often enter the surrounding environments through different media. Chow et al. (2023) noted that some of the peaks observed in their studies coincide with the pesticide spraying season in one of the catchment areas. Similarly, a study by Dabrowski et al. (2014) highlighted that runoff, spray drift, and leaching contribute to the movement of pesticides into areas where they are not needed. This is one of the ways pesticides can enter our environment. Du Preez et al. (2005) indicated that pesticides, including atrazine, can reach surface waters via redeposition after volatilisation and precipitation (Chow et al., 2023; Dabrowski et al., 2014; Du Preez et al., 2005).

Instances in which smallholder farmers apply these pesticides with minimal training or regulatory oversight increase the risk of overapplication, spillage, or unsafe disposal (Isgren & Andersson, 2020; Maja, 2022; Rother et al., 2008). Furthermore, using veterinary medicines such as antibiotics and antiparasitics, which can come from animals and enter the environment through manure spreading, runoff, or direct waste, increases the amount of micropollutants in the environment (Delgado et al., 2023; Udebuani et al., 2023).

### Domestic and urban sources

Urban and peri-urban households contribute to anthropogenic pollutants through widespread usage and disposal of over-the-counter and prescription pharmaceuticals (Magagula et al., 2022), as well as household insecticides and herbicides that are normally DIY, for example, vinegar (acetic acid) (Dougoud et al., 2019). Often, expired or unused medications or household insecticides and herbicides are flushed into toilets or discarded into household trash, thereby introducing them into sewage systems, landfills, or wastewater channels (Huici et al., 2017; Madikizela et al., 2020; Mogajane et al., 2024), resulting in their occurrence in ecosystems. Glyphosate is one of the herbicides that pose a risk to human health, especially to those living near farming areas (Gillezeau et al., 2019; Myers et al., 2016, 2022).

Insecticides used indoors and herbicides used in gardens can also be mobilised by rainwater runoff or atmospheric dispersal, particularly in high-density settlements with limited drainage systems (Adeyinka et al., 2019). These practices are often unregulated and driven by a lack of public awareness, the absence of take-back schemes, and inadequate policy on pharmaceutical waste management.

### Pharmaceutical manufacturing and hospitals

Pharmaceutical manufacturing plants and healthcare institutions (e.g. hospitals and clinics) are point sources of chemical pollution (Larsson, 2014). These facilities generate effluents that may contain active pharmaceutical ingredients (APIs) and formulation by-products. Discharges may result from manufacturing processes, equipment cleaning, disposal of unused or expired products, or patient excretion within healthcare settings (Malimba et al., 2024).

In South Africa, current regulations do not mandate API-specific effluent limits, and pre-treatment of hospital wastewater is generally not required. This regulatory gap allows APIs to enter municipal wastewater systems directly. A study done by Mahlaba et al. (2022) also highlights the lack of national legislation governing the disposal of medical waste for patients once they leave healthcare settings or hospitals. Maseko (2014) highlighted that some healthcare facilities do not have a satisfactory system for the segregation of medical waste or controlled disposal of expired or surplus medications due to inappropriate storage facilities, as well as insufficient training of medical staff on waste handling.

### Wastewater treatment plants (WWTPs)

Municipal WWTPs are critical nodes in the transport of both pharmaceuticals and pesticides from human use and urban runoff into the broader environment. The majority of WWTPs installed in South Africa were initially built to remove conventional pollutants such as solids, nutrients, and pathogens. State-of-the-art technologies such as oxidation, activated carbon filtration, or membrane filtration, which are necessary for removing trace amounts of organic pollutants, are often missing (Matesun et al., 2024; Rother, 2016).

Pharmaceuticals typically enter these systems through human excretion, improper disposal, and hospital discharges. For example, Magagula et al. (2022) discovered pharmaceuticals such as metformin (for diabetes) and antiretrovirals for HIV disease in WWTPs due to improper disposal via sinks or toilets. Commercial farms that use veterinary services also contribute to pharmaceutical pollution. Gulwako et al. (2023) noted that the improper disposal of veterinary products can also lead to environmental pollution. Similarly, Rother (2016) identified that improper disposal may cause pesticide residues to be transported to the water supply through produce washing, urban runoff, or infiltration from surrounding soils. Once they enter, contaminants remain through conventional treatment steps and are discharged through effluent or biosolids.

Furthermore, most plants also face high hydraulic loads, recurrent machinery breakdowns, or irregular monitoring. This increases the chances of ineffective treatment (Abafe et al., 2018).

### Informal settlements and non-sewered sanitation systems

Low-income neighbourhoods in informal settlements without proper sewage systems rely on pit latrines, mobile toilets, bucket systems, or the open dumping of wastewater, which creates pollution pathways (Chumo et al., 2023; Hermanus & Andrew, 2018; Makaudze & Gelles, 2015; Muanda et al., 2020; Shoniwa & Thebe, 2020). During rainfall events, overland flow can transport excreted pharmaceuticals through soils and shallow groundwater (Mamera, 2018). In addition, pest infestations have created a demand for cheap pesticides among street vendors, which results in poor sanitation and improper disposal of used bottles, ultimately leading to environmental pollution (Rother, 2016).

Greywater from bathing, washing, and cleaning, often contaminated with medical or personal care products, is commonly discharged into nearby yards, storm drains, or water bodies without treatment (Hermanus & Andrew, 2018; Horn et al., 2022b). These diffuse sources are challenging to monitor or regulate, yet they contribute significantly to cumulative pollution. The lack of infrastructure is further compounded by poor solid-waste management, minimal public education, and the absence of targeted sanitation policies for pharmaceutical waste in such areas.

## Review of field sampling and laboratory analysis methods for pesticide and pharmaceutical studies in South Africa

Detecting and quantifying pesticides and pharmaceuticals in the environment requires field sampling and appropriate analytical techniques for each compound. This section reviews the standard methods used in South Africa for collecting and analysing samples for pesticide and pharmaceutical contamination studies in different environmental matrices, including water, sediment, air, aquatic animals, and marine life (fish).

### Field sampling methods

Grab sampling has been the most used method mentioned in South African studies for sampling water and sediments. The convenience and cost-effectiveness of this method, along with its ability to provide accurate point-in-time data, make it the preferred method when easy access is unavailable or an urgent sampling need arises. For example, Dabrowski et al. (2002) used this method to quantify pesticide spikes in the Lourens River after rainfall and allowed them to identify the link between contamination and runoff. Similarly, Schulz (2001) used it to quantify pesticide drift from orchard catchments of the Western Cape, while Gakuba et al. (2019) and Matongo et al. (2015a) used grab samples to study river water and sediment for organochlorine and pharmaceutical residues. Archer et al. (2018, 2020, 2021) and Holton et al. (2023) also used grab samplers in numerous studies at various wastewater treatment works across South Africa.

While this approach does offer convenience, its most significant drawback is that it provides only snapshot data at a single point in time. This can miss temporary spikes in pollution or changes that happen throughout the day. Given this, grab sampling studies also need to account for time and location and include provisions for obtaining representative data.

Grab sediment samples can also provide information on how pollutants like pesticides and prescription drugs accumulate over time. Researchers such as Gakuba et al. (2019) and Mehlhorn et al. (2023) used grab sediment samplers and core devices to analyse the types of pollutants collected from the riverbed and estuary of the Umgeni River in KwaZulu-Natal and from Richards Bay in the Western Cape.

Passive sampling has also emerged as an important method among scientists in South Africa. This method of sampling has the benefit of mitigating the limitations of grab sampling by providing time-integrated observations. For example, Rimayi et al. (2019) in Gauteng province and Davies et al. (2024) in the Western Cape province used Chemcatcher® passive samplers in some rivers to monitor emerging contaminants over time. Passive sampling can distinguish low-concentration pollutants that may go undetected with grab sampling methods. As a result, passive sampling provides an ideal method for long-term observation and trend identification. Amdany et al. (2014) made use of a Semipermeable Membrane Device (SPMD) to identify long-lasting organic pollutants able to dissolve in water in water systems around Johannesburg. Similarly, Veludo et al. (2022) used passive air samplers to measure airborne pesticide levels in agricultural areas of the Western Cape. However, environmental factors such as flow rate and biofouling may impact results and require calibration. Furthermore, the use of passive samplers requires higher wait times for sample collection, which may delay real-time decision-making. Moreover, the polymer materials used in passive samplers may not attract and adsorb polar and non-polar compounds equally effectively, resulting in a loss of evidence (Greenwood et al., 2009).

### Sample preparations and laboratory analysis techniques

Solid-phase extraction (SPE) and solid-phase microextraction (SPME) were the most commonly used methods of sample preparation in laboratories in South Africa for concentrating pesticides and pharmaceuticals from environmental sample matrices. SPE and SPME were essential for accurately detecting trace amounts of pesticides and pharmaceuticals in samples such as water, fish, and sediments. Sigonya et al. (2022) used SPE and LC–MS/MS to detect pharmaceutical residues in water near Durban. Similarly, Matongo et al. (2015b), Ojemaye and Petrik (2022), and Ademoyegun et al. (2020) used SPE to study contaminations in rivers, fish, and sludge.

Dalvie et al. (2005) used SPME, a solvent-free method, and compared it with SPE and ELISA (enzyme-linked immunosorbent assay). Their findings suggested it was more cost-effective for rural pesticide screening. While SPE is practical and versatile for analyte recovery, it is labour-intensive and requires large volumes of solvent, making it expensive for rural labs. On the other hand, SPME simplifies the extraction process but may yield lower recoveries for polar compounds. Overall, the choice of extraction method in South Africa is determined by the target analytes, the sample type, and the availability of lab facilities.

### Quick, easy, cheap, effective, rugged, and safe (QuEChERS)

While solid-phase extraction (SPE) and solid-phase microextraction (SPME) have been the techniques used for sample preparation in South Africa, QuEChERS sample preparation in pesticides has also been used. This method is also useful because it can detect residues at ng/kg levels, which are far below the maximum residue levels allowed in foods. Similarly, the modified QuEChERS method was used to quantify organochlorine pesticide residues from fatty biological substances, like fish muscles, fat tissues, and South African coral (Buah-Kwofie & Humphries, 2019). This work also demonstrates the applicability of this method to both aquatic and terrestrial life. The comparison of the new QuEChERS-MISPE and the traditional QuEChERS-dSPE method was conducted by Mabunda et al. (2024) to determine the DDT levels in vegetables from South African Limpopo marketplaces. The results of this work indicate that the new QuEChERS-MISPE method performed better at target molecule identification, resulting in lower detection levels. Although this method is cost-effective on its own, it also has limitations related to complexity, matrix effects, and higher labour-intensive costs.

### Gas chromatography-mass spectrometry (GC–MS) and liquid chromatography-tandem mass spectrometry (LC–MS/MS)

These are also standard analytical instruments used by scientists to identify pesticides and pharmaceutical contaminants in South Africa. Semivolatile and volatile organochlorines are best suited for GC–MS, as stated by Ademoyegun et al. (2020) and Gakuba et al. (2019). LC–MS/MS, however, targets heat-labile compounds like antibiotics, antiretrovirals, and anti-inflammatories. LC–MS/MS was used by Abafe et al. (2018) to quantify antiretroviral drugs in influent and effluent water at wastewater treatment plants in KwaZulu-Natal, and the results indicated that removal was ineffective. Ojemaye and Petrik (2022) also used LC–MS/MS to quantify pharmaceuticals and PPCPs (PPCPs) in coastal waters in Cape Town and the risk faced by the ecosystem in the study area. While these methods provide high sensitivity, the operation also requires proficient people and up-to-date apparatus, as well as high-quality control. The use of LC–MS/MS by South African scientists demonstrates their skill improvement as they conduct tests analysing various substances.

Another method for describing the heterogeneous biological activity of chemicals from environmental samples is bioassays. Researchers have applied this method in mammalian cell-based and recombinant yeast oestrogen screening (YES) bioassays to measure the effects of pesticides and pharmaceutical residues that disrupt hormones. Mutengwe et al., 2016a, b) applied bioassays to screen foodstuffs for pesticide-induced estrogenicity, demonstrating the utility of functional endpoints in pollution analysis. Unlike other methods, which cannot identify the impact of specific compounds, bioassays are important for assessing overall toxicity and offer cheaper options or additional support for chemical analysis alone. They prove most useful for preliminary screening and risk prioritisation, as demonstrated in water and food chain exposure studies in South Africa. To be of maximum utility, however, bioassay results need to be instrumentally validated.

## Review of detected pesticides and pharmaceuticals in South Africa and their impacts

Various studies conducted across South Africa have reported widespread contamination of sediments, surface waters, estuaries, air, and marine biota with pharmaceuticals and pesticides. Table 1 (pharmaceuticals) and Table 2 (pesticides) provide an overview of where and how much specific micropollutants were detected across different provinces of South Africa, highlighting their environmental spread due to human activities. The table also lists the usual methods for finding and measuring the levels of these micropollutants, which come from agricultural wastewater treatment plants, landfill runoff, and household sources (Fig. 1).

**Table 1:** Concentration of selected pharmaceuticals detected in South Africa

Pharmaceuticals	Method of analysis	Concentration	Location	Reference
Caffeine, carbamazepine, diclofenac, illicit drugs, personal care products, etc	SPE + UHPLC-MS/MS	Caffeine: up to 31 µg/L; carbamazepine: 2.1 µg/L; diclofenac: 1.6 µg/L	Eerste River, Stellenbosch, Western Cape	Archer et al. (2023)
Pharmaceuticals and personal care products (PPCPs)	LC–MS/MS	0.01–0.89 µg/L	False Bay, Cape Town	Ojemaye and Petrik (2022)
Antiretroviral drugs (nevirapine, efavirenz)	LC–MS/MS	0.02–1.46 µg/L	WWTPs in KwaZulu-Natal	Abafe et al. (2018)
Ibuprofen, diclofenac, naproxen	LC–MS/MS	Up to 1.23 µg/L	Msunduzi River, KZN	Agunbiade and Moodley (2016)
Caffeine, carbamazepine, sulfamethoxazole	LC–MS/MS	Caffeine: 0.03–2.3 µg/L	Umgeni River, KZN	Agunbiade et al. (2014)
Estrogenic compounds (estrone, ethinylestradiol)	ELISA, Bioassay	EE2: up to 19.7 ng/L	Pietermaritzburg WWTP	Manickum and John (2014)

**Table 2:** Concentration of selected pesticides detected in South Africa

Pesticides	Method of analysis	Concentration	Location	Reference
Terbuthylazine, tebuconazole, carbendazim, dimethomorph, chlorpyrifos, etc.	LC–MS/MS, LC-HRMS	< LOQ to 1.8 µg/L (seasonal variation)	Agricultural catchments, Western Cape	Davies et al. (2024)
Chlorpyrifos, terbuthylazine, cypermethrin, lambda-cyhalothrin, etc.	LC-HRMS	0.001—> 1 µg/L (seasonal variation)	Western Cape agricultural watersheds	Chow et al. (2023)
Atrazine, simazine, alachlor, metolachlor, butachlor	LC–MS/MS	Atrazine: not detected (nd) to 66 ng/g dry weight (dw), simazine: nd to 158 ng/g dw, alachlor: nd to 48 ng/g dw, metolachlor: nd to 94 ng/g dw, and butachlor: nd to 9 ng/g dw	False Bay, Cape Town	Ojemaye et al. (2022)
Phthalates and OCPs	GC–MS	ND – 1.7 µg/L (water); up to 2.5 µg/kg (sediment)	Loskop Dam, Mpumalanga	Mathapelo et al. (2021)
Chlorpyrifos, terbuthylazine, cypermethrin, endosulfan (35 + pesticides)	GC–MS	ng–µg range per wristband (compound-specific)	Agricultural areas, Limpopo & Western Cape	Fuhrimann et al. (2021)
Chlorpyrifos, endosulfan, cypermethrin, terbuthylazine	GC–MS/MS	Air: up to 310 pg/m^3^; soil: up to 12.4 µg/kg, dust: up to 0.6 µg/g	Agricultural sites, Limpopo & Western Cape	Degrendele et al. (2022a)
DDT, DDE, endosulfan, etc	GC–MS	0.23–4.8 µg/g lipid (amphibian tissue)	Conservation areas, Limpopo & Mpumalanga	Wolmarans et al. (2021)
Endosulfan, DDT, aldrin, DBP, DEHP	GC–MS	Water: up to 1.7 µg/L; Sediment: up to 2.5 µg/kg	Loskop Dam, Mpumalanga	Mathapelo et al. (2021)
Organochlorines (DDT)	GC–MS	0.23–4.8 µg/g (tissue)	Malaria regions: Limpopo & Mpumalanga	Wolmarans et al. (2021)
Atrazine, chlorpyrifos, endosulfan, glyphosate, DDT, etc	GC–MS, LC–MS, Bioassays	Up to 2.5 µg/L (atrazine); 1.8 µg/L (endosulfan)	Multiple freshwater systems, SA (Western Cape, Mpumalanga, KZN, Limpopo)	Horak et al. (2021)
Terbuthylazine, cypermethrin, chlorpyrifos, metolachlor, tebuconazole, etc	LC–MS/MS, LC-HRMS	Up to 1.2 µg/L (seasonal, post-rainfall)	Three agricultural catchments, Western Cape	Curchod et al. (2020)
Alachlor, metolachlor, atrazine, simazine, etc	SPE + GC–MS	Up to 1.2 µg/L (river water); detectable in tap water	Letsitele, Lomati & Vals-Renoster catchments, South Africa	Machete and Shadung (2019)
DDT, HCH, aldrin, endosulfan, PCBs	GC-ECD	OCPs: up to 4.3 µg/kg; PCBs: up to 3.6 µg/kg	Umgeni River bank soil, KwaZulu-Natal	Gakuba et al. (2019)
Organochlorine pesticides (endosulfan, DDT, HCH)	GC-ECD	1.4–5.3 µg/kg (sediment)	Hartbeespoort Dam, NW	Rimayi et al. (2018)
Organophosphate and organochlorine pesticides	GC-ECD, GC–MS	0.11–3.25 µg/kg (sediments)	uMngeni River, KZN	Gakuba et al. (2019)
DDT, aldrin, dieldrin, heptachlor, endrin	GC-ECD	Up to 0.94 µg/L (surface water)	Buffalo River, Eastern Cape	Yahaya et al. (2017)
DDT, DDE, endosulfan	GC-ECD	1.1–4.2 µg/g lipid (crocodile fat tissue)	iSimangaliso Wetland Park, KwaZulu-Natal	Buah-Kwofie et al. (2018b)
Phthalate esters (DBP, DEHP)	GC–MS	0.1–1.9 µg/L	WWTPs in the Western Cape	Olujimi et al. (2012)
Bisphenol A, phthalates	GC–MS	BPA: 0.15–0.67 µg/L; DEHP: 0.32–1.21 µg/L	Venda and Western Cape	Fatoki et al. (2010)

*LC–MS/MS* liquid chromatography-tandem mass spectrometry, *LC-HRMS* liquid chromatography/high-resolution mass spectrometry, *SPE* solid-phase extraction, *UHP LC–MS/MS* ultra-performance liquid chromatography-mass spectrometry, *GC–MS/MS* gas chromatography-mass spectrometry/mass spectrometry, *GC–MS* gas chromatography-mass spectrometry, *LC–MS* liquid chromatography-mass spectrometry, *GC-ECD* gas chromatography-electron capture detector, *ELISA* enzyme-linked immunosorbent assa, *LOQ* limit of quantification, *ND* not detected, *µg/L* micrograms per litre, *µg/g* micrograms per gramme, *pg/m*^*3*^ picograms per cubic metre, *µg/kg* micrograms per kilogramme, *ng–µg* nanogram-microgram, *µg/g* micrograms per gramme, *EE2* 17α-ethynylestradiol, *BPA* bisphenol A, *OCPs* organochlorine pesticides, *PCBs* polychlorinated biphenyls, *DEHP* di(2-ethylhexyl) phthalate, *SA* South Africa, *KZN* KwaZulu-Natal, *NW* North West

Surface water and sediments in KwaZulu-Natal frequently contain acidic pharmaceuticals like diclofenac, ibuprofen, and naproxen. These pharmaceutical residues were observed in the Agunbiade and Moodley (2016) and Ndungu (2016) studies from the Umgeni and Msunduzi rivers, with elevated concentrations downstream of treatment works. Gumbi et al. (2017) also demonstrated, using GC–MS, that acidic pharmaceutical residues are not effectively removed in municipal treatment systems. The same types of pharmaceutical pollutants were observed in Cape Town marine waters, where Ojemaye and Petrik (2022) detected various personal care products and pharmaceuticals that posed ecological risks, particularly for invertebrates and fish in False Bay.

Contamination by pesticides is also widespread. Rimayi et al. (2018) reported various emerging contaminants, including p-chloroaniline, a pesticide found in the Umgeni River estuary and the catchment of the Hartbeespoort Dam. Gakuba et al. (2019) also established the presence of organochlorine pesticides (OCPs), including aldrin, o,p-DDE, p,p′-DDE, endrin, DDT, and Mirex, in deposits found in Umgeni River waters and sediment samples. Meanwhile, Ademoyegun et al. (2020) identified similar compounds in sewage sludge collected from wastewater treatment plants in the Raymond Mhlaba local municipality in the Eastern Cape, South Africa. Among the various compounds detected by Gakuba et al. (2019) and Ademoyegun et al. (2020), Aldrin, Mirex, Endrin, and many other OCPs are banned in South Africa. The detection of these OCPs in the studies highlights issues with regulations and indicates that banned pesticides may still be present in the South African environment, possibly due to residues left by previous applications (Veludo et al., 2022; Horak, 2021).

Endocrine-disrupting compounds (EDCs) such as phthalates, bisphenol A, and phenolic compounds were also widely detected. Fatoki et al. (2010) detected phthalate esters and alkylphenols in Venda water systems at concentrations higher than those in other studies and correlated them to reproductive and developmental disorders. Furthermore, Manickum and John (2014) noted poor removal efficiency of oestrogenic EDCs at the wastewater treatment plant in Pietermaritzburg, which posed risks to aquatic life.

The increasing levels of these pollutants have been associated with health challenges for humans. In 2024 alone, there were 890 foodborne pesticide poisoning notifications in all provinces of South Africa, which, in one case, resulted in the deaths of more than six children, of which the mortality was directly attributed to poisoning by terbufos (The Presidency, 2024). This aligns with reviews indicating that pesticide toxicity remains a contributor to child and adolescent mortality in South African urban centres (Davies et al., 2023). Prinsloo et al. (2025) tracked the ingestion-related deaths, and their study indicated that foodborne illness is increasingly becoming the cause of death for both children and adults in many parts of South Africa. Overall, Prinsloo et al. (2025) noted that pesticides were the most common cause of foodborne-related poisoning and death in children (42%) and adults (29%). Although pathways of food contamination by pollutants vary, studies indicate that exposure is indirectly due to contaminated water used in food processing.

Additionally, findings from a study in the Eastern Cape indicated that exposure to pesticides was associated with congenital disabilities, especially among women who stayed and laboured on farms (Felisbino et al., 2024). Similarly, a study by Fuhrimann et al. (2022) that examined silicon wristbands worn by children and their guardians in agricultural households in the Western Cape found that participants were exposed to pesticides, with children experiencing higher levels of exposure than adults. Another study by Degrendele et al. (2022a) examined air and soil samples from two agricultural areas in the Western Cape, South Africa. They confirmed chlorpyrifos exposure via soil absorption, specifically in areas with the most prominent pesticide use. In a study in the Western Cape Province, Chetty-Mhlanga et al. (2021) found a connection between children’s exposure to pesticides at work and their behaviour and thinking. This connection was stronger in neurocognitive performance, especially among children who performed jobs such as fruit picking or worked near sprayed fields. A broader environmental risk study conducted by Fuhrimann et al. (2021) confirmed the presence of hazardous pesticide mixtures in the Western Cape farming region, highlighting risks to both the environment and human health. The findings show the high risks of occupational and environmental exposure to toxic chemicals.

Screening for antiretroviral drugs was also conducted by Abafe et al. (2018) at the entry and discharge points of a wastewater treatment plant in KwaZulu-Natal. Their findings raised concerns about environmental harm and the spread of drug-resistant bacteria. In another related study, Tucker et al. (2022a, 2022b) examined wastewater treatment plants in Cape Town. Their findings reveal that while the treatment reduced most antibiotic-resistant genes and bacteria, the treated water still contained some resistant genes and bacteria that could pose a health and environmental risk. The study also found high levels of microbial resistance in samples collected near wastewater treatment plant discharge points. This suggests that these plants can both help stop and also contribute to the spread of antibiotic resistance. The persistence of pharmaceuticals that promote antimicrobial resistance in effluent samples from treatment plants further underscores the limitations of such installations and their failure to treat pharmaceutical waste.

Reviewed studies have indicated that concentrations of micropollutants in river water and biota, such as atrazine (detected to be up to 2.5 µg/L by Horak et al. (2021)) and endosulfan (detected to be up to 1.8 µg/L by Horak et al. (2021), Mathapelo et al. (2021), and Rimayi et al. (2018)), exceeded internationally accepted thresholds. For example, the EU environmental quality standards (EQS) for endosulfan are 0.01 µg/L and 2.0 µg/L for atrazine in drinking water according to the EU guidelines (European Parliament and Council, 2013). Furthermore, chlorpyrifos, a pesticide still registered in South Africa, is restricted in the EU due to neurotoxicity concerns, yet has been detected in South African water systems. Some studies, e.g. Chow et al. (2023), Curchod et al. (2020), Davies et al. (2024), and Horak et al. (2021), have indicated that the concentration of chlorpyrifos exceeds environmental quality standards.

## Regulations on pesticides and pharmaceuticals pollution in South Africa

South Africa has established regulatory systems to address the environmental and health risks posed by pesticides and pharmaceuticals. Separate institutions enforce regulations under multiple pieces of legislation. Although there is considerable headway in registration, import control, and safety testing, significant gaps remain in enforcement, surveillance, and suitability for current chemical and environmental issues. Compared to best practices in Europe (EU), Australia, America (US), Switzerland, and select Asian countries, South Africa’s regulations require modernisation and integration into a cohesive process.

### Legislative framework for pesticides and pharmaceuticals in South Africa

The primary regulation of pesticides in South Africa is under the Fertilisers, Farm Feeds, Agricultural Remedies, and Stock Remedies Act No. 36 of 1947 (Department of Agriculture, 1947), managed by the Department of Agriculture, Land Reform, and Rural Development (DALRRD). Regulation of agricultural remedy registration and control is legal, although the 1947 regulation is outdated and lacks provisions for modern chemical formulations and public health pesticides. The Foodstuffs, Cosmetics and Disinfectants Act No. 54 of 1972 (Department of Health, 2024) complements this regulation by establishing the maximum permitted levels of pesticides in foodstuffs. Regulation is complemented by South Africa’s 2010 adoption of a Pesticide Management Policy to ensure human and environmental safety through improved legislation and institutional capacity.

Pharmaceuticals are managed by the Medicines and Related Substances Act No. 101 of 1965 (Department of Health, 2025) and regulated by the South African Health Products Regulatory Authority (SAHPRA). Although the Act is responsible for drug efficiency, quality, safety, and registration issues, it does not address the entry of pharmaceuticals into the environment. Wastewater treatment plants (WWTPs) must comply with general water discharge parameters in South Africa, yet current regulations do not account for the occurrence limits of pharmaceutical residues. General environmental legislation, e.g. the National Water Act and the National Environmental Management Act, provides an enabling framework for water quality but not for emerging substances such as antibiotics, hormones, and antiretrovirals (Adom & Simatele, 2022; Ngqwala & Muchesa, 2020).

### Banned and restricted pesticides and pharmaceuticals in South Africa

South Africa has attempted to limit the use of harmful pesticides by using DALRRD’s lists of banned and restricted substances (Table 3). Aldrin, endosulfan, and DDT are included, although DDT is allowed only under WHO guidelines for controlling malaria. The country is also a signatory to the Stockholm Convention on Persistent Organic Pollutants (POPs), which requires the phasing out of listed hazardous substances. However, its implementation is weak, and highly hazardous pesticides (HHPs) continue to be used and detected in the environment. There were 192 HHPs registered in South Africa, of which more than one-third were prohibited within the EU (Unpoison South Africa, 2023).

**Table 3:** List of selected banned and restricted pesticides in South Africa based on the Department of Agriculture (2017); Unpoison South Africa, 2023)

Pesticide	Status	Regulatory reference
Aldicarb	Banned	Government Notice No. 862, 29 July 2016
Aldrin	Banned	Government Notice No. R. 384, 25 February 1983
Arsenic Compounds	Restricted	Use on plant material (except citrus) banned in 1983; Government Notice No. R. 384, 25 February 1983
Atrazine	Restricted	Withdrawn from use on heavy clay soils in 1977; industrial use withdrawn on 31 March 1995
Azinphos-ethyl	Banned	Government Notice No. 862, 29 July 2016
Binapacryl	Banned	Government Notice No. 862, 29 July 2016
Camphechlor (Toxaphene)	Banned	Government Notice No. 862, 29 July 2016
Captafol	Banned	Government Notice No. 862, 29 July 2016
Chlordane	Banned	Government Notice No. R. 834, 26 August 2005
Chlordimeform	Banned	Government Notice No. 862, 29 July 2016
Chlorobenzilate	Banned	Government Notice No. 862, 29 July 2016
Chlorpyrifos	Restricted	Banned for household and garden use in 2010; Government Notice No. R. 375, 14 May 2010
2,4-D (dimethylamine salt)	Restricted	Banned in parts of Camperdown, Pietermaritzburg, and Richmond; aerial application banned in KwaZulu-Natal in 1991; Government Notice No. R. 2370, 27 September 1991
2,4-D Esters	Restricted	Withdrawn from all agricultural uses in the Western Cape in 1980; banned in KwaZulu-Natal in 1991; Government Notice No. R. 2370, 27 September 1991
2,4-DB (dichlorophenoxybutyrate sodium salt)	Restricted	Banned in parts of Camperdown, Pietermaritzburg, and Richmond; aerial application banned in KwaZulu-Natal in 1991; Government Notice No. R. 2370, 27 September 1991
Dicamba	Restricted	Banned in parts of Camperdown, Pietermaritzburg, and Richmond; aerial application banned in KwaZulu-Natal in 1991; Government Notice No. R. 2370, 27 September 1991
DDT (dichlorodiphenyltrichloroethane)	Restricted	Banned in 1983, except for malaria vector control by the government; Government Notice No. R. 384, 25 February 1983
Dibromochloropropane	Banned	Government Notice No. 862, 29 July 2016
Dieldrin	Banned	Government Notice No. R. 384, 25 February 1983
Dinoseb	Banned	Government Notice No. 862, 29 July 2016
Dinitro-ortho-cresol (DNOC)	Banned	Government Notice No. 862, 29 July 2016
Endosulfan	Banned	Government Notice No. 853, 26 October 2012
Terbufos	Restricted	Its use is strictly limited to licenced agricultural professionals, and it is on the Department of Agriculture’s list to be banned by 2024
Endrin	Banned	Government Notice No. 862, 29 July 2016

The informal markets still sell prohibited pesticides such as terbufos, resulting in tragic health consequences. In 2024, six children lost their lives in Soweto as a result of eating snacks that were contaminated with terbufos (Mutsila, 2025). Such incidents expose weaknesses in regulation and enforcement, as well as public awareness.

In South Africa, there is no complete list of pharmaceuticals that are banned or taken off the market, but SAHPRA has removed certain non-steroidal anti-inflammatory agents (NSAIDs; Table 4) and addictive substances due to their abuse by the public (Swartz et al., 2018). For example, Davies et al. (2024) detected methaqualone (mandrax) in Hex, Grabouw, and Piketberg agricultural catchments in the Western Cape, which is one of the pharmaceuticals that are banned in South Africa. Although no specific research has examined the impact of mandrax on the South African ecosystem, its presence in the environment still poses various environmental risks (Bornman & Bouwman, 2012). However, mandrax has been found to be persistent and poses ecological risks to aquatic and ecological systems in countries like Brazil and Mexico (Luna et al., 2017; Mannarino, 2013; Prado Flores & Martínez Ibarra, 2019). Environmental pollution is worsened by weak policies and the absence of take-back programmes or organised methods for disposing of expired or unused medications, which are often flushed down the toilet or thrown in regular trash.

**Table 4:** Banned and restricted pharmaceuticals in South Africa

Pharmaceutical name	Status	Regulatory reference
Pholcodine-containing medicines	Banned	SAHPRA—April 2023
Benylin paediatric syrup	Banned	SAHPRA—April 2024
Colistin (antimicrobial)	Restricted	Requires Section 21 approval for use
Codeine (in high doses)	Restricted	Controlled under Schedule 6 regulations
Thalidomide	Restricted	Requires strict regulation due to teratogenic effects
Certain NSAIDs, such as Alclofenac and Mibefradil	Banned	Withdrawn due to safety concerns
Phenolphthalein	Banned	Withdrawn from the market due to carcinogenicity concerns
Sibutramine (Reductil)	Banned	Withdrawn due to increased cardiovascular risk
Ephedrine	Restricted	Controlled under Schedule 6 regulations
Methaqualone (Mandrax)	Banned	Classified as an illegal substance
Phenacetin	Banned	Withdrawn due to nephrotoxicity and carcinogenicity
Dipyrone (Metamizole)	Banned	Withdrawn due to the risk of agranulocytosis

### Comparative review of pesticides and pharmaceutical regulations: South Africa vs. international standards

This section compares the pesticide and pharmaceutical frameworks in South Africa with those of other leading international standards, including the USA, Switzerland, Australia, and selected Asian countries. The section aims to highlight regulatory gaps in South African regulatory frameworks or systems. The comparison included legislative systems, risk assessment processes, monitoring programmes, standards of protection for workers, and the use of sustainable substitutes.

### Legislative frameworks and regulatory oversight

South Africa’s primary legislation, the Fertilisers, Farm Feeds, Agricultural Remedies, and Stock Remedies Act (Act No. 36 of 1947), is administered by the Department of Agriculture, Land Reform, and Rural Development (DALRRD). Although this act establishes a foundational regulatory framework for pesticide registration, it does not include updated provisions to address contemporary environmental risks, such as mandatory environmental risk assessments (ERAs), routine monitoring, and disposal safeguards.

In comparison, the European Union adopts comprehensive pesticide legislation through its regulation No. 1107/2009 and the Water Framework Directive (2000/60/EC), both supported by the European Food Safety Authority (EFSA) (European Parliament and Council, 2009; European Parliament & Council, 2000). In Europe, pharmaceutical regulation is under Directive 2001/83/EC, which stipulates that there should be an ERA for new pharmaceuticals (European Medicines Agency, 2006). In the USA, pesticides are regulated under the Federal Insecticide, Fungicide, and Rodenticide Act (FIFRA) as well as the Food Quality Protection Act (FQPA). 21 CFR, part 25 of the Code of Federal Regulations (U.S. FDA, 1998), focuses on environmental oversight for pharmaceuticals in the USA. Australia operates under the Agricultural and Veterinary Chemicals Code Act, which is administered by the Australian Pesticides and Veterinary Medicines Authority (APVMA). This body is responsible for evaluating the safety, efficacy, and environmental impact of chemical products before approval (Australian Government, 2023). Switzerland conducts frequent re-evaluations of pesticide registrations through its Federal Office for Agriculture (FOAG); these re-evaluations closely align with EU restrictions. In Asia, countries like Japan and South Korea have enacted ERA requirements while improving their regulatory capacity (FAO, 2021; Korea Rural Economic Institute, 2022; Ministry of Agriculture, Forestry, and Fisheries of Japan, 2022; Ministry of Environment of Korea, 2020).

### Pharmaceutical regulation and environmental protection

Regulatory controls of pharmaceuticals in the EU (via the European Medicines Agency, EMA) and the USA (via the Food and Drug Administration, FDA) also provide oversight mechanisms. In the EU, automatic environmental risk assessments (ERAs) apply to all new applications for marketing authorisation for pharmaceuticals, and they consider potential impacts on the ecological environment (European Parliament and Council, 2009; European Medicines Agency, 2006; Zinken et al., 2024). Similarly, the FDA in the USA also insists upon drug-use and drug-disposal-pattern-based assessments of the environment (U.S. FDA, 1998; Walter & Mitkidis, 2018). Additionally, pharmaceutical take-back programmes, such as the EU’s Extended Producer Responsibility programme and various US drug enforcement strategies, aim to reduce the inappropriate disposal of unused pharmaceuticals.

South Africa lacks binding environmental regulations for pharmacovigilance, often because there is little coordination among various programmes, which generates fragmented data collection (Mehta et al., 2014). The majority of active pharmaceutical ingredients lack complete ERA requirements, are often treated as confidential data, and are subject to little public access to valuable environmental data (Moeti et al., 2023). This regulatory exemption has direct implications for environmental and public health outcomes and calls for the strengthening of environmental laws on pharmaceuticals (Moodley & Suleman, 2019; Suleman & Gray, 2017).

### Risk assessments, prohibitions, and monitoring gaps

The pesticide registration model of South Africa usually allows a pesticide to remain in circulation unless it has been proven to be harmful to the environment (Rother, 2010; Utembe, 2019). The EU and Switzerland, on the other hand, preemptively ban high-risk pesticides. According to Unpoison South Africa (2023), two hundred and twenty-two (222) highly hazardous pesticides (HHPs) are currently registered for use in South Africa, of which 30 are entirely banned, 16 are partially banned, and 176 remain in use with no strict restrictions. Fifty-seven are banned in the EU, and only eight are banned in both the EU and South Africa. Hazardous pesticides such as paraquat, atrazine, and chlorpyrifos, which are prohibited in the EU and Switzerland, are still permitted for “strict use” in South Africa. This highlights the weak precautionary stance of South Africa’s pesticide regulations.

South Africa primarily monitors pesticide residues in export products, typically destined for markets with stricter regulations, such as the EU. Monitoring for products intended for domestic consumption, as well as for water bodies, soils, and sediments, is less frequent (Mutengwe et al., 2016a, b). Furthermore, occupational pesticide exposures are inadequately addressed (Roosli et al., 2022). In contrast, the EU exerts more stringent monitoring on its imported products for domestic consumption through the pesticide residue-monitoring programme of the European Food Safety Authority (European Food Safety Authority et al., 2024). The USA conducts similar strict monitoring through the Pesticide Data Program (PDP). Australia has the National Residue Survey (NRS), and Switzerland regularly checks its products as part of its rules for plant protection products, which require reassessment of active substances and the inclusion of environmental monitoring data in their reviews. These structured frameworks are largely absent in South Africa.

In South Africa, there is no national programme to monitor pharmaceutical residues in the environment.

### Occupational health, public safety, and sustainable alternatives

There is a high risk of pesticide poisoning in South Africa due to weak enforcement of informal pesticide sales, low public awareness, and insufficient occupational safety training (Chetty-Mhlanga et al., 2021). Although the South African Poison Information Helpline provides 24-h assistance, limited public awareness and underutilisation hinder its impact on pesticide exposure response and surveillance. Furthermore, enforcement of regulations on personal protective equipment (PPE) and pesticide handling and disposal practices is often inadequate. It is unclear whether DALRRD provides training on PPE and pesticide handling and disposal practices.

In contrast to the above, the EU, Switzerland, and the USA each enforce compulsory certification and safety training for pesticide handlers. For example, in the USA, there is a worker protection standard enforced through the Environmental Protection Agency (EPA) (U.S. Environmental Protection Agency, 2015). In Australia, licencing and structured risk assessments and communication are strictly required; Japan has implemented strict labelling standards (Ministry of Agriculture, Forestry, and Fisheries of Japan, 2022), while South Korea integrates pesticide education into their agricultural extension services (Korea Rural Economic Institute, 2022). These safeguards are largely absent in South Africa, thereby increasing the risk of harm and exposure to farmworkers and rural communities.

In terms of sustainable alternatives, despite being a signatory to the Stockholm Convention (United Nations Environment Programme, 2001), which requires countries to develop action plans for sustainable alternatives to the most harmful pesticides, South Africa’s efforts to implement its Integrated Pest Management (IPM) have been limited (Srinivasan et al., 2022). On the other hand, the EU, Switzerland, and Australia have fully embedded IPM into their national strategies. Countries like Japan and South Korea have also promoted biopesticides as sustainable alternatives (FAO, 2021; Ministry of Environment of Korea, 2020).

## Conclusion and recommendations

### Conclusion

**Fig. 2:**
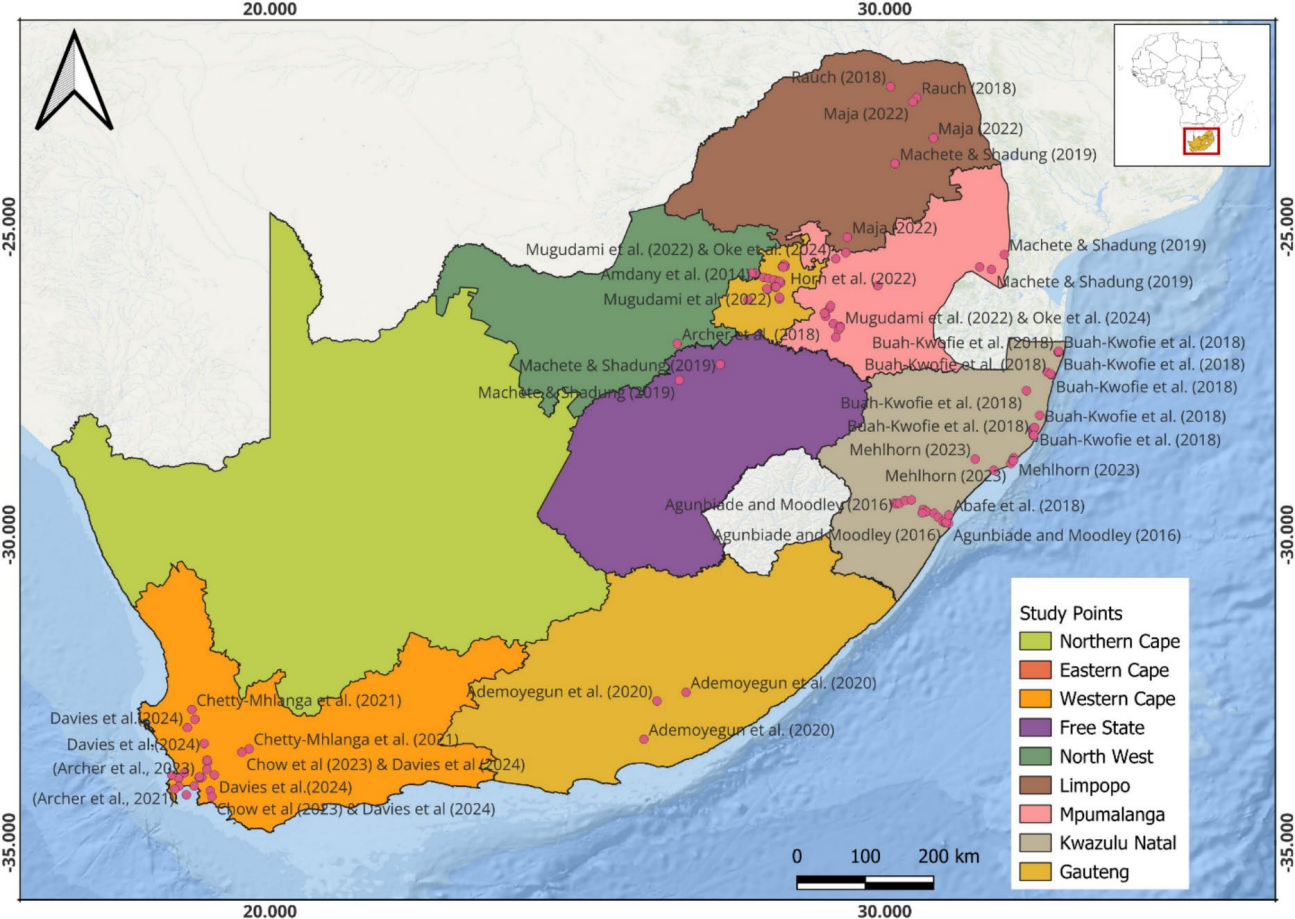
Map showing the location of various studies done across South Africa on pharmaceuticals and pesticides. The maps indicate that between 2010 and 2025, which is our review timeline, more studies have been carried out in the Western Cape, KwaZulu-Natal, and Gauteng

Our review indicates that pesticide and pharmaceutical pollution pose significant environmental and public health risks in South Africa, particularly in agricultural areas, those close to wastewater discharge points, and densely populated areas, as observed in the reviews. Reviewed studies between 2010 and 2025 (with most of the studies in Western Cape, KwaZulu-Natal, and Gauteng: Fig. 2) have found various micropollutants in surface water, sediments, marine life, and air, often at levels above levels considered safe by international standards. However, there are still areas in South Africa with limited research on the issue (the Northern Cape, Free State, Northwest, and Eastern Cape). This calls for further studies to understand the impact of these toxic and persistent compounds in South African environments.

South Africa’s existing regulatory frameworks, led by DALRRD and SAHPRA, still lack critical elements, including mandatory environmental risk assessments (ERAs), structured monitoring programmes, and national take-back programmes. The continued registration, use, and abuse of banned and restricted substances like terbufos, methaqualone (mandrax), and chlorpyrifos illustrate these gaps. In contrast, jurisdictions such as the EU, USA, Switzerland, Australia, and selected Asian countries implement precautionary bans, routine re-evaluation, and extensive residue-monitoring systems in the environment and for food items intended for both domestic and local consumption.

Occupational exposures among farmworkers, informal pesticide sales, and unregulated pharmaceutical sales and disposal remain primary sources of risk to both human and environmental health. Furthermore, the slow adoption of the integrated pest management scheme and limited investment in analytical and monitoring initiatives further compound these challenges.

### Recommendations

To tackle these compound issues, the following comprehensive strategies are suggested:

1. *Regulatory reform, monitoring frameworks, and take-back schemes*: South Africa will need to revise and modernise its core pesticide and pharmaceutical legislation to mandate environmental risk assessments (ERAs) and align substance bans with the World Health Organisation’s (WHO) and the Food and Agriculture Organisation’s (FAO) highly hazardous pesticide (HHP) criteria. It is recommended to establish monitoring programmes for pesticides and pharmaceuticals across various environments (e.g. water, soil, air, marine life, and food) and to create national take-back initiatives, coordinated among regulatory agencies such as SAHPRA, DALRRD, DWS, and DFFE, to prevent overlap.
2. *Data infrastructure, laboratory capacity, and research investment*: A centralised, open-access database should be developed to host monitoring data categorised into contaminant type, location, and environmental matrix. In addition, the South African government, through various ministries, should invest in expanding and equipping regional laboratories with advanced instruments such as LC–MS/MS and GC–MS, and in establishing quality assurance protocols. This will improve research capabilities, helping to assess ecological risks, examine combined exposure routes, and study the effects of anthropogenic pollutants on the environment and public health in South Africa.
3. *Occupational safety, public awareness, and sustainable alternatives*: SAHPRA and DALRRD should implement national safety training and certification programmes for pesticide and pharmaceutical handlers in line with international standards like the EPA’s Worker Protection Standard. Public awareness campaigns must educate rural populations, farming communities, and schools on safe handling, disposal, and alternatives to hazardous substances.

## Data Availability

This study does not include primary data. All analysis is based on previously published literature.
